# Supplementary material for: Assembly Characteristics and Influencing Factors of the Soil Microbial Community in the Typical Forest of Funiu Mountain
Source: Microorganisms. 2024 Nov 18;12(11):2355. doi: 10.3390/microorganisms12112355 (PMC11596301; doi:10.3390/microorganisms12112355)
Supplement: Supplementary file 1 [file microorganisms-12-02355-s001.zip › microorganisms-3282370-supplementary.pdf]

Table S1. Basic characteristics of the experimental site.

	Larix gmelinii (LG)	*Quercus aliena* var. *acutiserrata* (QA)	*Quercus aliena* var. *acutiserrata* + *Pinus armandii* (QAPA)
Location	111°40′37″E, 33°38′2″N	111°40′58″E, 33°37′55″N	111°40′32″E, 33°40′25″N
Altitude (m)	1303.05	1343.78	1482.47
Slope (°)	28	31	35
Aspect	NE	NE	NE
SBD	0.8	0.85	0.89
Canopy density	0.5	0.85	0.8

Table S2. The litter and soil properties in different forests.

	LG	QA	QAPA
LC (g/kg)	213.52±6.2B	203.49±14.75B	253.41±8.08A
LL (g/kg)	373.7±10.29A	320.21±3.35A	347.29±7.58A
LOC (g/kg)	283.42±16.11B	294.46±34.44B	393.89±7.75A
LTN (g/kg)	14.61±0.89B	14.83±1.35B	18.95±0.35A
LTP (g/kg)	0.75±0.03A	0.66±0.05A	0.76±0.02A
LCN	15.19±1.21A	14.14±0.76A	13.46±0.62A
LLN	26.49±1.9A	23.61±2.58AB	18.37±0.49B
LON	19.49±0.44A	19.49±0.74A	20.85±0.57A
LCP	289.81±14.74A	310.5±17.67A	335.39±16.75A
LLP	504.95±18.95A	504.52±36.57A	458.95±18.9A
LOP	379.01±14.81B	436.14±35.13AB	519.81±18.81A
LNP	19.55±0.9B	22.12±1.01AB	24.94±0.66A
SWC	13.09±1.04B	21.23±0.9A	13.16±0.95B
pH	4.97±0.08A	5.2±0.11A	4.94±0.07A
SBD	0.8±0.02B	0.85±0.02AB	0.89±0.02A
SOC (g/kg)	11.42±0.91B	15.85±1.86B	26.87±2.5A
STN (g/kg)	0.8±0.06B	1.24±0.14B	1.75±0.18A
STP (g/kg)	0.24±0.01A	0.25±0.01A	0.24±0.01A
SCN	14.27±0.18B	12.75±0.15C	15.56±0.2A
SCP	49.02±4.2B	64.68±6.97B	112.29±6.58A
SNP	3.44±0.29C	5.05±0.53B	7.25±0.48A
AN (mg/kg)	17.43±1.76B	29.99±3.15A	29.29±2.35A
NN (mg/kg)	0.9±0.17A	0.91±0.11A	0.68±0.17A
DOC (mg/kg)	99.21±9.84B	137.42±14.54B	215.39±16.15A
DON (mg/kg)	14.83±1.61B	25.35±2.35A	26.25±2.16A
AP (mg/kg)	11.97±2.2A	3.74±1.06B	4.11±0.32B
RCN	6.93±0.51B	5.45±0.32C	8.33±0.31A
RCP	15.33±5.45B	59.28±11.24A	54.55±4.9A
RNP	2.3±0.8B	10.69±1.86A	6.6±0.55A
MBC (mg/kg)	158.31±12.98B	207.83±22.65B	304.88±21.93A
MBN (mg/kg)	12.08±1.36B	15.28±1.95B	23.75±1.44A
MBP (mg/kg)	8.78±0.6A	9.95±0.84A	11.19±0.75A
MCN	13.57±0.62A	14.05±1.07A	12.84±0.46A
MCP	18.03±0.75B	20.61±0.99B	27.2±0.72A
MNP	1.35±0.08B	1.52±0.12B	2.14±0.09A

Notes: LC: cellulose, LL: lignin, LOC: litter organic carbon, LTN: litter total nitrogen, LTP: litter total phosphorus, LCN: LC/LTN, LLN: LL/LTN, LON: LOC/LTN, LCP: LC/LTP, LLP: LL/LTP, LOP: LOC/LTP, LNP: LTN/LTP, SWC: soil water content, SBD: soil bulk density, SOC: soil organic carbon, STN: soil total nitrogen, STP: soil total phosphorus, SCN: SOC/STN, SCP: SOC/STP, SNP: STN/STP, AN: soil ammonium nitrogen, NN: soil nitrate nitrogen, DOC: soil dissolved organic carbon, DON: soil dissolved organic nitrogen, AP: soil available phosphorus, RCN: DOC/DON, RCP: DOC/AP, RNP: DON/AP, MBC-soil microbial biomass carbon, MBN-soil microbial biomass

nitrogen, MBP-soil microbial biomass phosphorus, MCN: MBC/MBN, MCP: MBC/MBP, MNP: MBN/MBP.

Table S3. Comparison of network topology under different forests.

	LG	QA	QAPA
Nodes	203	177	166
Edges	261	216	141
Average degree (avgK)	2.571	2.441	1.699
Average clustering coefficient (avgCC)	0.063	0.076	0.063
Average path distance (GD)	5.179	5.993	2.319
Modularity	0.808	0.809	0.952
